# Live Imaging and Interactome Analysis of Zika and Chikungunya Viral RNAs Via Dual-Action Aptamer Tag

**DOI:** 10.21203/rs.3.rs-6992950/v1

**Published:** 2025-07-07

**Authors:** Joyce Jose, Anastazia Jablunovsky, Anoop Narayanan

**Affiliations:** The Pennsylvania State University; The Pennsylvania State University; The Pennsylvania State University

## Abstract

Arthropod-borne, positive-sense RNA viruses encompass many human pathogens posing significant health threats worldwide. Understanding intracellular dynamics and host-factor interactions of the viral genome is essential for devising effective antiviral strategies. We engineered dual-labeled Zika and chikungunya viruses incorporating a Mango-II aptamer for RNA pulldown and real-time imaging, achieving high-resolution imaging of flavivirus RNA in live cells. Tandem imaging with viral replication proteins revealed intracellular dynamics of replication complexes and translating RNA of these viruses. We identified > 1,000 high-confidence host interactors of viral RNA and corroborated 355 hits from existing data sets. Spliceosome factor SFPQ was identified as a common interactor decreasing RNA production in both viruses when depleted. Intracellular colocalization of SFPQ-ZIKV RNA was confirmed through super-resolution imaging of infected cells. Thus, we demonstrate Mango-II aptamer tagging as an innovative strategy for identifying spatiotemporal dynamics and virus-host interactions of viral genomes in positive-strand RNA viruses.

## Introduction

Viruses from the *Flaviviridae* and *Togaviridae* families efficiently infect vertebrate and arthropod cells by hijacking various host factors to translate, replicate, and package their positive-strand (+) genomic RNA. Orthoflaviviruses such as Zika virus (ZIKV), dengue virus (DENV), and Powassan virus (POWV) from *Flaviviridae*, as well as alphaviruses like chikungunya virus (CHIKV) and Eastern equine encephalitis virus (EEEV) from *Togaviridae*, are known to cause severe diseases in humans, including polyarthritis, hemorrhagic fever, and fatal encephalitis.^1–3^ Zika virus infections have been reported in over 80 countries, with the Americas experiencing a large outbreak from 2015–2016 that directly caused an increased incidence of microcephaly in infants.^4,5^ An estimated 390 million dengue virus infections occur annually, with approximately 21,000 deaths attributed to the disease.^6,7^ Since 2005, over 2 million cases of chikungunya (CHIKV) infection have been reported worldwide, with outbreaks occurring in over 110 countries across Africa, Asia, Europe, and the Americas.^8^ Major CHIKV outbreaks are currently ongoing in the Americas, with Brazil reporting 71,578 cases, while La Réunion, France, has reported over 47,500 cases and 12 deaths as of May 4, 2025 (WHO). Alphaviruses and Orthoflaviviruses are small, enveloped, spherical virions packaging a single copy of their (+) strand RNA genome encapsidated by several copies of a capsid protein within a lipid bilayer embedded with transmembrane envelope glycoproteins arranged in icosahedral symmetry. Both virions enter host cells via receptor-mediated endocytosis and undergo fusion of the viral and host membranes in the late endosome, releasing their viral genomic RNA (vgRNA) into the cytoplasm.

In infected cells, the orthoflavivirus vgRNA is translated by ribosomes on the endoplasmic reticulum (ER) into a polyprotein, which is cotranslationally translocated onto the ER membrane and cleaved by host and viral proteases into three structural proteins [capsid (C), premembrane (prM), and envelope (E)] as well as seven nonstructural proteins (NS1, NS2A, NS2B, NS3, NS4A, NS4B, and NS5).^9^ Replication of vgRNA is mediated by the RNA-dependent RNA polymerase (RdRp) NS5 from the replicase complex found in virus-induced replication organelles, which are formed by ER membrane invaginations where the positive-sense vgRNA and its negative-sense complement create a pseudo-circular double-stranded intermediate.^10,11^ Nascent (+) RNA that exits the replication compartments is designated for translation, replication, or packaging by the C protein to form virions; however, the spatiotemporal distribution of vgRNA is not well understood.^9,10,12^ A subset of the nascent (+) RNA molecules forms subgenomic flavivirus RNA (sfRNA) fragments through incomplete degradation by host exoribonucleases, contributing to pathogenesis through interaction with host proteins.^13,14^ The packaged nucleocapsid of the vgRNA-C protein complex buds into the ER lumen, where it acquires a prM/E envelope, forming the immature virus, which traffics through the secretory pathway, undergoing maturation, which includes low pH-mediated prM/E rearrangement and a furin cleavage of prM before finally exiting the cell as mature virions.

Unlike orthoflaviviruses, the released alphavirus vgRNA is translated by polysomes into two nonstructural polyproteins (nsP1–3 and nsP1–4), which are processed by the nsP2 protease into four nonstructural proteins, nsP1, nsP2, nsP3, and nsP4.^15,16^ Initial RNA replication occurs in virus-induced replication spherules on the plasma membrane, followed by late replication on endosomal and lysosomal membranes that form cytopathic vacuole type I (CPV-I) vesicles at the perinuclear region.^17–20^ Following replication through a dsRNA intermediate mediated by the RdRP nsP4 from the replicase complex, a subgenomic 26S RNA is formed from the (−) RNA, which encodes the structural polyprotein CP-E3-E2-6K-E1 processed to form the capsid protein (CP), Envelope proteins E3, E2, E1 and 6K ion channel protein, as well as a transframe protein (TF) formed by ribosomal frameshifting on 6K.^21^ The N-terminal CP is released from the structural polyprotein by autoproteolytic cleavage, the remaining structural proteins are translocated and processed in the secretory pathway, and trimeric spikes of E2/E1 heterodimers are transported to the plasma membrane. The viral genomic RNA (vgRNA) assembles into nucleocapsid cores through the interaction of CP with a packaging signal and subsequently buds from the plasma membrane through a specific interaction with the cytoplasmic domain of E2, releasing virions.^22–24^

Cryogenic electron microscopy (cryo-EM) and electron tomography studies have unveiled the structural aspects and life cycle stages of orthoflaviviruses and alphaviruses. Near-atomic cryoEM structures of mature and immature orthoflaviviruses, including ZIKV, DENV, TBEV, Binjari virus, Spondweni virus,^24–29,^ and alphaviruses such as CHIKV, Sindbis virus (SINV), Mayaro virus, WEEV,^31–34^ have revealed details of the structural organization of virions. Resin-embedded transmission electron microscopy (TEM) and cryoelectron tomography studies have shown the organization of virus-induced membrane modifications, cytoskeletal rearrangements, and assembly sites, demonstrating extensive interactions with the host in orthoflavivirus- and alphavirus-infected cells.^10,35–39^

Investigating host interactions with viral RNA using mass spectrometry and live and fixed imaging has expanded in recent years, and leveraging tools from gene knockout studies has enabled the identification of relevant interactors critical for the virus life cycle.^40^ Pulldown studies targeting the viral RNA of ZIKV, DENV, CHIKV, and SINV revealed a complex network of interactions with known and non-canonical RNA binding proteins (RBPs).^41–45^ Notably, previous work has relied on UV or chemical crosslinking to capture RBPs, thereby obscuring the distinction between stable and transient interactions.^46^ Yet identifying RBPs through the pulldown of RNA in its native context has not been feasible, which is necessary to determine the complex interactions of vgRNA during infection. The dynamic nature of viral proteins and virions in the context of virus infection has been explored using super-resolution and live microscopy^47–49^; however, the lack of effective tools has impeded the investigation of vgRNA dynamics in live infected cells. Intracellular RNA visualization has been established using fluorescence *in situ* hybridization (FISH) in orthoflaviviruses and alphaviruses; however, this technique is limited to fixed-cell imaging, lacking temporal resolution.^50–56^ Live-cell imaging of RNA can be achieved by RNA aptamers, which bind to fluorescent proteins or dyes to enable imaging. This strategy was used effectively in a replicon system of the flavivirus tick-borne encephalitis virus; however, the 24x aptamer repeats required for sufficient signal, combined with the steric bulk of the fused binding protein, have prevented application in infectious viruses.^57^ More recently, the RNA aptamer Broccoli was used to image alphavirus RNA from SINV in live cells; however, this approach was unable to achieve single-molecule resolution^58^

The Mango aptamer, developed in 2014 by Dolgosheina et al., is a small (< 60 nt) G quadruplex structure that binds to the fluorogenic dye thiazole orange 1-biotin (TO1-B) to generate a strong green fluorescent signal.^59^ In addition to the high-quality imaging properties, Mango aptamers are compatible with streptavidin column pulldown, allowing both intracellular localization and protein interactome data to be gathered from the same construct.^60,61^ Second-generation Mango-II-IV aptamers have been developed with stronger fluorescence and improved binding affinity for TO1-B, resulting in stable arrays of Mango-II triplets for enhanced imaging capabilities.^62,63^ We used the dual-action Mango-II triplet tag for high-resolution live imaging and proteomics of native viral genomic RNA in two unrelated RNA viruses, ZIKV and CHIKV, to investigate their infectious life cycles. We resolved the trafficking of viral RNA in association with modified ER for ZIKV and endo-lysosomal vesicles in CHIKV. Among several interacting partners identified and validated, the nucleic acid binding protein SFPQ (splicing factor proline and glutamine rich) emerged as a host interactor of both ZIKV and CHIKV vgRNA, which showed a significant impact on viral RNA replication upon knockdown in infected cells. Subsequent imaging revealed that SFPQ colocalizes with vgRNA in the perinuclear region in ZIKV. This study presents a two-pronged approach to investigating viral RNA, applicable to diverse positive single-stranded RNA viruses.

## Results

### Generation of Mango-II aptamer-tagged viruses

**Figure 1:**
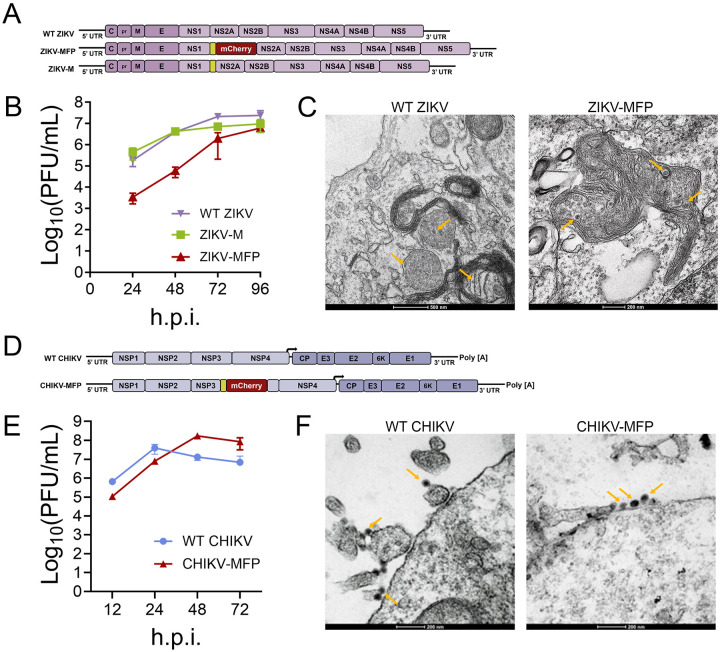
Design and engineering of Mango-II aptamer-tagged ZIKV and CHIKV. (A) Schematic of viral genomes of WT ZIKV, ZIKV-MFP, and ZIKV-M indicating the location of Mango aptamer and mCherry tags. (B) Graph of growth kinetic analysis of WT ZIKV, ZIKV-MFP, and ZIKV-M obtained from infected JEG-3 cells. (n = 3). (C) Micrograph of TEM analysis from Huh7.5 cells infected with WT ZIKV and ZIKV-MFP. Yellow arrows indicate virus particles. The scale bar is shown below. (D) Schematic of the organization of WT CHIKV and CHIKV-MFP viral genome RNA indicating the position of Mango II aptamer and mCherry tags. (E) Graph of growth kinetic analysis of viruses produced by WT CHIKV and CHIKV-MFP from U2OS cells. (n = 3). (F) Micrograph of TEM analysis from Huh 7.5 cells infected with WT CHIKV or CHIKV-MFP. Yellow arrows indicate viral particles. The scale bar is shown below. Tag sequences and locations are shown in Table S1.

Advancements in live-cell compatible RNA imaging techniques have been significantly hindered by the existing tags, which are bulky, and the challenges in identifying non-lethal insertion sites in RNA, limiting the ability to establish viral RNA dynamics in live cells for most positive single-stranded RNA viruses. Based on the relatively small size (159 nt) and the ability to generate single-molecule resolution signal in optical microscopy, we selected the fluorogenic Mango-II aptamer triplet tag for insertion into the genome of representative members of the *Flaviviridae* (ZIKV) and *Togaviridae* (CHIKV) families. We amplified a Mango-II cassette from the pLenti-mCherry-Mango II × 24 plasmid (Addgene, #127587) containing three TO1-B binding sites for inserting into cDNA clones of CHIKV and ZIKV.^63^ We inserted the Mango-II triplet sequence, devoid of stop codons, into the 5’ end of ZIKV NS2A, a site that has been shown to tolerate a large fluorescent protein tag (ZIKV-FP).^64^ (Table. S1) We engineered two ZIKV clones expressing Mango-II, first with a single Mango-II triplet inserted directly onto NS2A as a peptide tag consisting of 53 amino acids (ZIKV-M), and the second where the DNA sequence corresponding to the Mango-II triplet is linked to an mCherry fluorescent protein gene, generating a dual-labeled ZIKV expressing Mango-II-mCherry fluorescent protein-tagged NS2A (ZIKV-MFP) (Fig. 1A) (Fig. S1). We performed growth kinetic analysis for wild-type ZIKV (WT-ZIKV), ZIKV-M, and ZIKV-MFP and determined the virus titer. The maximum titer of WT-ZIKV was 2.43×10^7^ PFU/ml, comparable to ZIKV-M, which reached a maximum titer of 9.60×10^6^ PFU/ml at 96hours post infection (h.p.i.) (Fig. 1B) ZIKV-MFP was attenuated, showing ~ 2 log reduction compared to WT-ZIKV at early 24 and 48 h.p.i. time points before reaching a maximum titer of 6.13 ×10^6^ PFU/ml at 96 h.p.i. (Fig. 1B). Transmission electron microscopy (TEM) was performed in WT ZIKV and ZIKV-MFP-infected human Huh7.5 cells to confirm the formation of viral particles (Fig. 1C). Convoluted membrane structures are observed in both samples, further confirming that Mango-II tag insertions support infection and can be used to obtain biologically relevant data.

To generate Mango-II tagged CHIKV, we used a cDNA clone of CHIKV strain 181/25, a gift from Naomi Forrester, University of Texas Medical Branch at Galveston, TX.^65,66^ Like ZIKV-MFP, we engineered a dual-labeled 181/25 cDNA clone containing a Mango-II triplet sequence linked to an mCherry fluorescent protein inserted into a flexible region of nonstructural protein 3 (nsP3), which has been previously shown to tolerate large insertions without affecting virus growth in other alphaviruses (CHIKV-MFP) (Fig. 1D).^67^. We performed growth kinetic analysis of WT CHIKV and CHIKV-MFP and quantified the production of infectious virus at indicated time points hours post infection by plaque assay. The WT CHIKV titer reached an average maximum of 4.00 ×10^7^ PFU/ml at 24 h.p.i., then decreased at later time points due to high pathogenicity and cell death (Fig. 1E). The CHIKV-MFP produced infectious virus to an average titer of 8.00×10^6^ PFU/ml at 24 h.p.i., which was comparable to that of WT CHIKV (Fig. 1E). The CHIKV-MFP titer continued to increase, reaching a peak of 1.73 4.00 ×10^8^ at 48 h.p.i.^68,69^. We also performed TEM analysis on CHIKV and CHIKV-MFP-infected human Huh7.5 cells to detect virus production. Viral particles were observed budding from the plasma membrane in both samples (Fig. 1F), confirming that virus assembly and release are unaffected by the insertion of tags in the CHIKV MFP.

### Mango-II imaging of single-stranded vgRNA is validated by FISH

**Figure 2:**
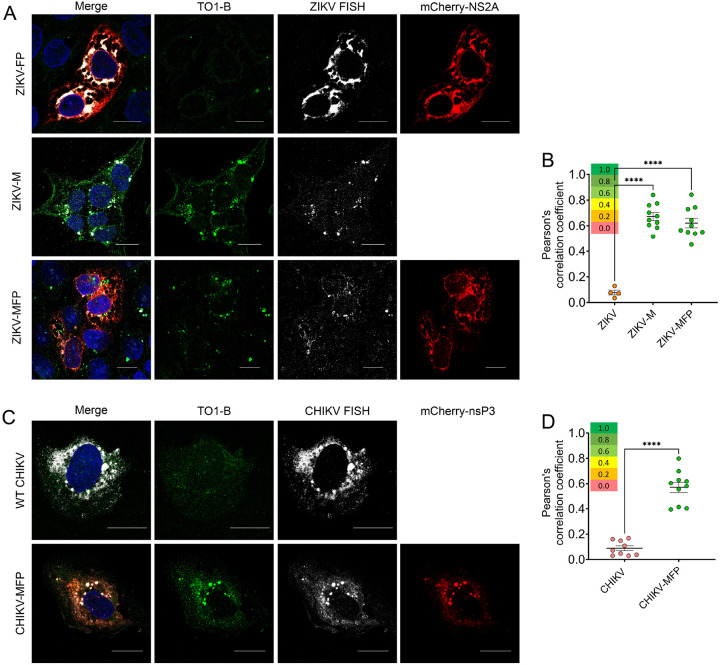
Mango-II tagged vgRNA colocalizes with positive-strand viral RNA in fluorescence in situ hybridization (FISH). (A) Fluorescent micrographs of fixed JEG-3 cells infected with fluorescent protein-tagged ZIKV-FP (mCherry-NS2A, red), ZIKV-MFP, or ZIKV-M and treated with ZIKV-specific FISH probes (far red, pseudo-colored white) followed by TO1-B staining (green) and Hoechst nuclear stain (blue). (B) Graph of Pearson’s correlation values showing colocalization of green (535 nm) and far-red (670 nm) channels for each micrograph in (A) (n=4 or 10; error bars represent mean ± SEM). (C) Fluorescent micrographs of fixed U2OS cells infected with fluorescent protein-tagged CHIKV-FP (mCherry-nsP3, red) or CHIKV-MFP and treated with CHIKV specific FISH probes (far red, pseudo-colored white) followed by TO1-B staining (green) and Hoechst nuclear stain (blue). (D) Graph of Pearson’s correlation values showing colocalization of green (535 nm) and far-red (670 nm) channels for each micrograph in (C) (n=10; error bars represent mean ± SEM). All scale bars in (A) and (C) correspond to 20 μm. Significance in (A) and (C) were calculated by One-way ANOVA using GraphPad Prism software. ****p < 0.0001.

During replication, both alphavirus and orthoflavivirus vgRNA enter a double-stranded (dsRNA) intermediate state as the negative-sense strand is generated and becomes the template for new positive-sense vgRNA molecules. To determine if the Mango-II tag can be detected in this dsRNA replication state or exclusively as single-stranded molecules, we designed FISH probes to target the single-stranded vgRNA specifically. Starting with ZIKV, human placental cells (JEG-3) were infected with ZIKV-WT, ZIKV-MFP, or ZIKV-M, then fixed at 36 h.p.i. and permeabilized. These cells were then treated with fluorescently tagged FISH probes targeting ZIKV (+) single-stranded RNA and subsequently with TO1-B and Hoechst stains and imaged using confocal microscopy. In the ZIKV-FP control, which contains no Mango-II tag, FISH detection showed RNA in association with NS2A along the ER and in large virus-induced perinuclear compartments (Fig. 2A top); however, there is minimal fluorescence in the green channel, showing that TO1-B does not bind to untagged vgRNA. Conversely, in ZIKV-M and ZIKV-MFP, highly dynamic punctate structures with green fluorescence were observed, representing TOI-B bound to the Mango-II aptamer tag on vgRNA Fig. 2A, middle, bottom). Pearson’s correlation analysis was performed by comparing the green (TO1-B) and far-red (FISH) channels, which revealed low overall colocalization in the negative control, the untagged virus, with an average coefficient of 0.112. In contrast, both tagged viruses, ZIKV-M and ZIKV-MFP, had average PC values over 0.600 (Fig. 2B). This confirms that Mango-II-tagged ZIKV constructs produce a sufficient signal for visualization and that the foci observed are consistent with the established localization of ZIKV vgRNA by FISH.

Next, we infected U2OS cells with CHIKV or CHIKV-MFP, fixed at 16 h.p.i., and permeabilized using 0.05% Triton X-100. The cells were then treated with FISH probes targeting CHIKV (+) single-stranded vgRNA, followed by TO1-B and Hoechst staining. In cells infected with WT CHIKV, we observed fluorescence from the FISH throughout the cytoplasm and in vesicular structures localized to the perinuclear region (Fig. 2C, top). In cells infected with CHIKV-MFP, green fluorescence from TO1-B is observed in the cytoplasm, perinuclear region, and on the plasma membrane, where early replication of alphaviruses occurs (Fig. 2C, bottom).^70^ Minimal fluorescence was observed in the green channel for the negative control WT CHIKV, indicating that TO1-B does not bind to the vgRNA of untagged WT CHIKV. Pearson’s correlation analysis showed that colocalization between FISH and TO1-B is high in CHIKV-MFP with an average Pearson’s correlation value of 0.57 compared to CHIKV-WT, which has minimal FISH/TO1-B colocalization with an average PC value of 0.09 (Fig. 2D). Therefore, the RNA localization detected by Mango in CHIKV is consistent with FISH, specific for the (+) single-stranded vgRNA.

### The Mango-II tag does not detect double-stranded RNA

**Figure 3:**
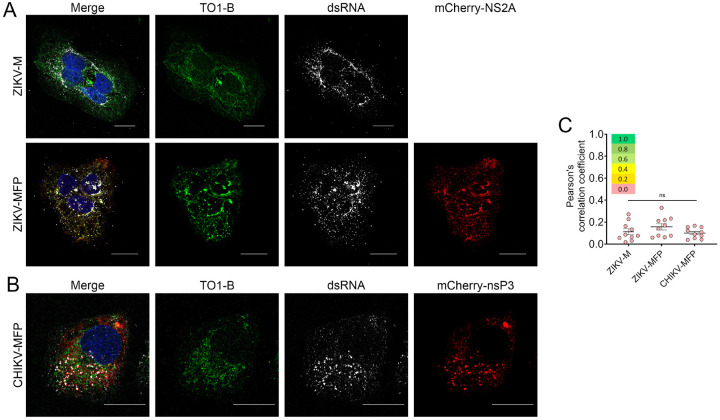
Mango-II imaging exclusively detects single-stranded vgRNA, and not dsRNA replication intermediate. (A) Fluorescent micrographs of JEG-3 cells infected with ZIKV-M (top) or ZIKV-MFP (bottom) fixed at 24 h.p.i. (B) Fluorescent micrograph of U2OS cells infected with CHIKV-MFP fixed at 16 h.p.i. Infected cells in (A-B) were treated with J2 dsRNA antibody (far red, pseudo-colored white) and TO1-B stain (green). All scale bars in (A-B) correspond to 20 μm. (C) Colocalization of green (535 nm) and far-red (670 nm) channels for (A-B) measured by Pearson’s correlation (n=10; error bars represent mean ± SEM). Significance was calculated by One-way ANOVA using GraphPad Prism software. n.s. p > 0.05.

To detect dsRNA replication sites, we performed an immunofluorescence assay (IFA) using an anti-dsRNA antibody, along with TO1-B staining, in cells infected with ZIKV-M or ZIKV-MFP (Fig. 3A). In both samples, green fluorescence is observed along the ER and accumulates in the perinuclear region. In contrast, dsRNA is observed in distinct puncta representing replication sites formed during ZIKV infection (Fig. 3A). Similarly, U2OS cells infected with CHIKV-MFP were fixed and treated with dsRNA antibodies. We detected dsRNA in distinct puncta, indicating sites throughout the cytoplasm, in contrast with Mango-II staining, which was more diffuse (Fig. 3B). Pearson’s correlation analysis determined that the colocalization between TO1-B and dsRNA was minimal, with all three tagged viruses showing average PC values below 0.20, indicating that the RNA visualized using the Mango-II aptamer tag does not detect replicating RNA in the double-stranded state (Fig. 3C).

### Live-cell imaging of ZIKV and CHIKV vgRNA using Mango-II aptamer tag

The primary advantage of using a fluorogenic aptamer tag is the ability to image vgRNA in live cells. Additionally, creating dual-labeled viruses that express a fluorescent protein-tagged viral protein allows for the convenient identification of viral protein-associated organelles and the relative stage of infection in infected cells. During ZIKV infection, the transmembrane NS2A protein localizes broadly to the ER membrane at early time points. As ZIKV infection modifies the intracellular membranes, NS2A localizes on the tubular ER membrane and, in large ER-associated vesicles, accumulates in the perinuclear region during late infection.^35^ In contrast, CHIKV nsP3 can be observed on the plasma membrane where early-stage (3–6 h.p.i.) replication and translation occur, as well as on endosomal membranes, lysosomes, and large type I cytoplasmic vesicles (CPV-I) during later time points.^19,71,72^

**Figure 4:**
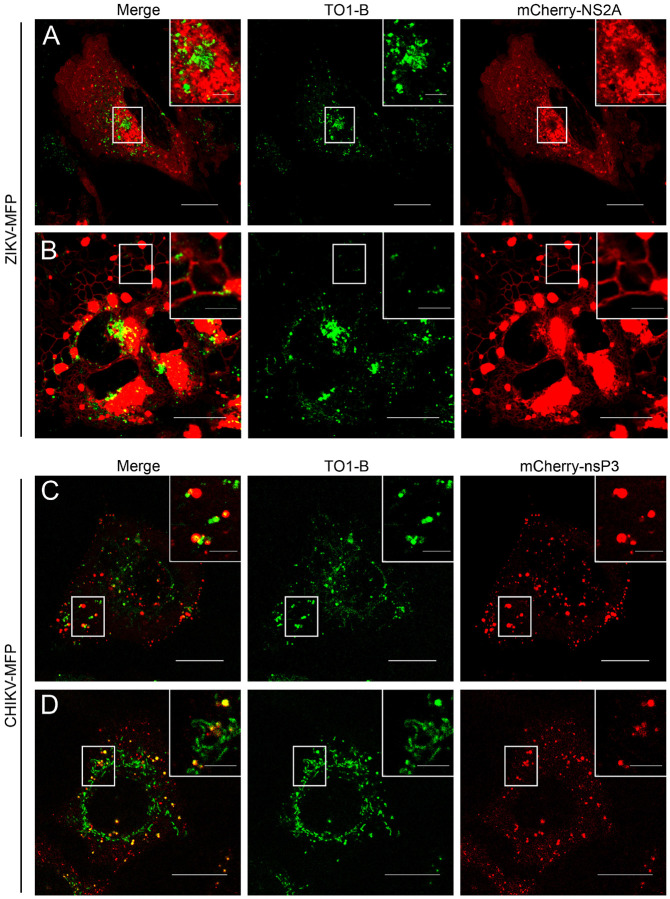
Live-imaging of vgRNA in ZIKV and CHIKV infected cells. (A-B) Representative images in merge (left) green (center) and red (right) channels from live-imaging experiments (Videos S1-S2) of ZIKV-MFP infected Huh7.5 cells stained with TO1-B. The white box indicates ROI shown enlarged in the top right corner. Scale bar indicates 20 μm. (C-D) Representative images in the merge (left) green (center) and red (right) channels from live-imaging experiments (Videos S3-S4) of CHIKV-MFP-infected U2OS cells stained with TO1-B. For (A, B, C and D), the white box indicates the ROI shown enlarged in the top right corner. The scale bar in full-size images indicates 20 μm, and the scale bar in zoomed ROI panels indicates 5 μm. See also Fig. S1.

Having established the ability of Mango-II to visualize RNA molecules in infected cells, we set out to image the spatial and temporal dynamics of vgRNA molecules relative to viral proteins and associated cellular organelles during active infection. We infected Huh7.5 cells with ZIKV-MFP and stained the cells with TO1-B at 72 h.p.i. Infected cells showed NS2A-FP associated with the entire ER network of flattened membrane sheets, known as cisternae, located near the nucleus and corresponding to the rough ER (Fig. 4A). In these cells, green foci indicating TO1-B bound vgRNA can be seen in distinct puncta (Fig. 4A, zoom) throughout the ER and in the perinuclear region. Notably, there is a large cluster of TO1-B-associated foci at the center of the perinuclear structure where NS2A and other viral proteins are known to accumulate. These RNA foci are not significantly colocalized with NS2A-FP (Fig. 4A, Zoom). Cells were also identified as representing tubular ER based on the red fluorescence of NS2A-mCherry localization (Fig. 4B). We observed vgRNA puncta moving along the tubule structures of the ER (Fig. 4B, Zoom). Large NS2A-containing vesicles were also detected to be associated with the modified ER (Fig. 4B, Zoom); however, the vesicles were largely devoid of TO1-B-bound RNA. Much of the NS2A in these cells accumulates at the perinuclear region of a distinctly kidney-shaped nucleus, a documented modification of ZIKV-infected cells (Figure C).^35^ Our results show, for the first time, that ZIKV vgRNA is highly dynamic in live cells and is associated with the ER membrane during infection.

To image CHIKV-MFP, we infected U2OS cells and stained the cells with TO1-B at 6–8 h.p.i. to evaluate the dynamics of vgRNA in relation to the labeled viral protein, nsP3-mCherry. Infected cells were identified by the red fluorescence of nsP3-mCherry localization on the plasma membrane where early replication and translation occur (Fig. 4C). The green fluorescence corresponding to TO1-B-bound CHIKV vgRNA was observed on the plasma membrane and the limiting membranes of cytoplasmic vesicles, indicating colocalization with endosomes, lysosomes, and CPV-I, and possibly membrane-less stress granules (Fig. 4C). The vgRNA was observed colocalizing with nsP3-containing vesicles, as well as on dynamic foci containing no nsP3 and large filamentous structures (Fig. 4C, zoom). We then imaged infected cells in the presence of LysoTracker, a cellular stain, and confirmed the colocalization between vgRNA-lysosomes and endosomes (Figure S3). In addition to vgRNA on lysosomes and nspP3-associated vesicles, we also observed cells containing vgRNA in filamentous formations throughout the cytoplasm (Figs. 4D). Notably, unlike ZIKV vgRNA, much of the CHIKV vgRNA was not associated with the ER membrane. Our data confirm the colocalization of alphavirus vgRNA and a mCherry-tagged viral protein, nsP3 in live cells and reveal interactions between vgRNA and nsP3-mCherry in real-time. A comparison of CHIKV with ZIKV vgRNA reveals marked differences in vgRNA localization and trafficking, shedding light on the functional diversity of these RNA viruses.

### Host interactome of ZIKV and CHIKV vgRNA using Mango-II tag and AP-MS

**Figure 5:**
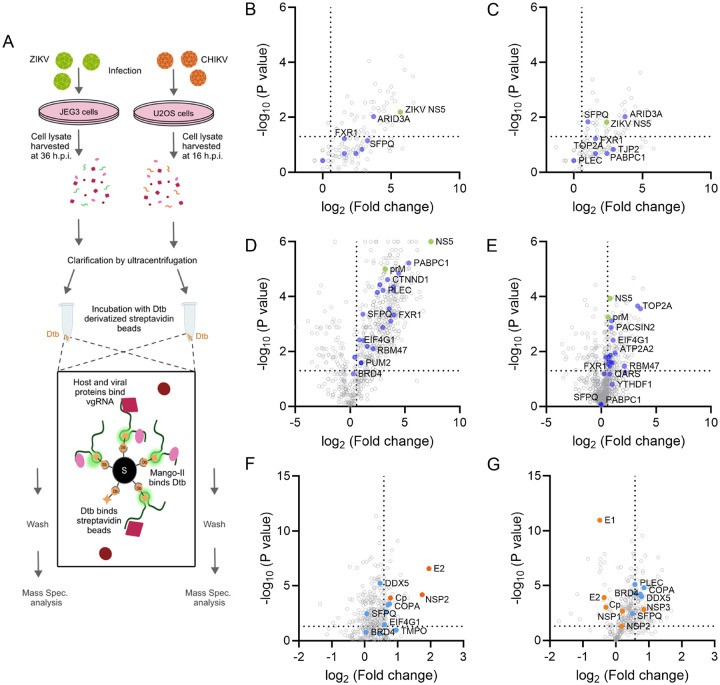
Pulldown of RNA from virus-infected cells using Mango-II aptamer tag. (A) Schematic illustration of pulldown protocol. Dtb = desthiobiotin and S = streptavidin bead. (B-C) Volcano plots showing enrichment of proteins pulled down by ZIKV-M vgRNA. (B) shows the P value and fold change over mock-infected cells, and (C) shows the P value and fold change over WT ZIKV-infected cells. (D-E) Volcano plots showing enrichment of proteins pulled down by ZIKV-MFP vgRNA. (D) shows the P value and fold change over mock-infected cells, and (E) shows the P value and fold change over ZIKV-infected cells. Purple circles indicate proteins of interest, and green circles indicate viral proteins. (F-G) Volcano plots showing enrichment of proteins pulled down by CHIKV-MFP vgRNA. (F) shows the P value and fold change over mock-infected cells, and (G) shows the P value and fold change over WT CHIKV-infected cells. Blue circles indicate proteins of interest, and orange circles indicate viral proteins. The threshold values (indicated by gray dashed lines) used for all volcano plots were a fold change >1.5 of and a P value of <0.05, as calculated by multiple t-test analysis using GraphPad Prism software. The technical replicates were n=3 for all samples. The total Mass spectrometry results are presented in Table S2. Comparison with the available literature data sets is shown in Table S3. See also Fig. S2.

We utilized the RNA pulldown ability of the Mango-II tag via affinity purification to determine the host protein interactome of Mango-II-tagged ZIKV vgRNA. We infected JEG-3 cells in 150 mm culture plates with WT ZIKV, ZIKV-M, ZIKV-MFP, or mock infection for 36 hours, then harvested the cell lysate, clarified by ultracentrifugation, and incubated with streptavidin resin derivatized with desthiobiotin to allow Mango-II aptamer binding (Fig. 5A). The bound resin was then washed and subjected to LC/MS using a Q-Exactive Plus mass spectrometer (Thermo Fisher Scientific). For ZIKV-M-infected cells, 138 proteins were enriched with fold change values ≥ 1.5 compared to mock, and 129 proteins were enriched compared to the negative control ZIKV-WT with no mango tag (Figs. 5B–C). The Mango-II pulldown of ZIKV-MFP had a greater abundance of total hits with 814 proteins enriched compared to mock and 393 proteins enriched over untagged WT ZIKV (Figs. 5D–E). Comparison of the datasets showed consistent results, with 180 out of 191 total ZIKV-M hits also detected in ZIKV-MFP. ZIKV NS5 was enriched in all Mango-tagged samples, indicating a strong interaction between the RdRP and vgRNA. Gene Ontology analysis was performed on the top 200 hits, revealing that RNA binding was the most enriched function, with a false discovery rate (FDR) of 1.27×10^−50^ (Figure S1). We also observed significant enrichment in ATP hydrolysis activity (FDR = 1.53×10^−25^), isopeptide bond association (FDR = 2.27×10^−25^), RNA splicing (FDR = 6.52×10^−15^), and host-virus interaction (FDR = 7.21×10^−10^), among others.

We performed a similar Mango-II RNA pulldown and AP-MS experiment using wild-type CHIKV (WT-CHIKV), CHIKV-MFP, and mock-infected U2OS cells to determine the host protein interactome of CHIKV vgRNA. Due to the initial low abundance of proteins from U2OS cells, the CHIKV samples were processed with peptide purification and labeling, followed by Nano-LC-MS/MS using an EASY-nLC 1200 HPLC system (SCR: 014993, Thermo Fisher Scientific), which increased the total number of detected proteins from 100 to 759. In CHIKV-MFP-infected cells, 275 proteins were enriched with fold change values ≥ 1.5 compared to mock, and 277 proteins were enriched compared to WT (Figs. 5F–G). Viral structural proteins CP, E1, and E2 were enriched over WT CHIKV-infected cells, indicating that these structural proteins interact directly with the viral RNA. Nonstructural proteins nsP1, nsP2, and nsP3 were also detected with significant enrichment values compared to mock-infected cells; however, only nsP3 was enriched with an average fold change of 7.09 over WT CHIKV. Among the host proteins identified, RNA binding was determined to be the most enriched function by GO analysis of the top 200 hits with an FDR of 9.63×10^−34^ (Figure S1), as observed in ZIKV AP-MS. We also observed significant enrichment in Intracellular non-membrane bound organelle association (FDR = 5.53×10^−22^), Isopeptide bond association (FDR = 2.19×10^−33^), and Nucleoplasm (FDR = 9.34×10^−18^), as well as enrichment in host-virus interaction (FDR = 2.39×10^−6^).

### Analysis of proteins interacting with both ZIKV and CHIKV vgRNA

**Figure 6:**
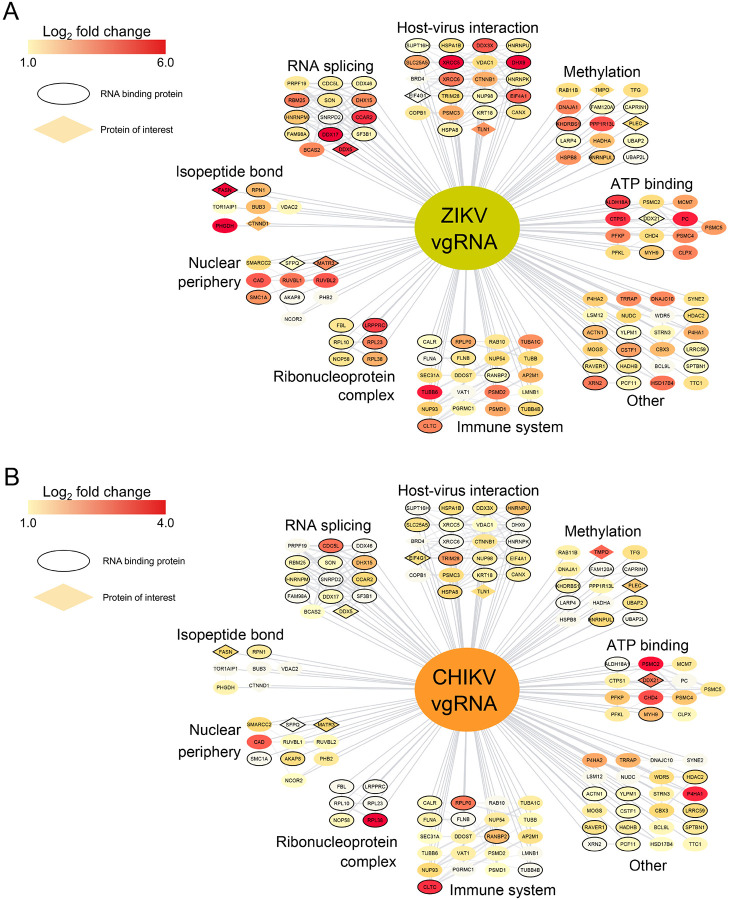
Overlapping hits from the vgRNA pulldown results. (A) Interactome map of ZIKV vgRNA with 132 high-confidence hits also identified by CHIKV pulldown. Color is mapped to the relative log_2_ of the fold change value, as indicated in the legend at the top left. (B) Interactome map of CHIKV vgRNA with 132 high-confidence hits also identified by ZIKV pulldown. Color is mapped to the relative log_2_ of the fold change value, as indicated in the legend at the top left. All protein clusters were determined by STRING enrichment in Cytoscape. The black border indicates RNA-binding proteins, and diamonds indicate selected proteins of interest.

We compared the MFP-infected enrichment over mock from ZIKV and CHIKV vgRNA pulldowns and identified 132 high-confidence overlapping protein hits (Fig. 6A–B). We performed a String analysis of protein-protein interaction and Gene Ontology (GO) enrichment on the list of common proteins using Cytoscape.^73^ The most significantly enriched molecular function was RNA binding (FDR = 2.71×10^−33^). However, non-canonical RNA binding proteins (RBPs) were also detected (Fig. 6). Virus-host interaction encompassed a large subgroup of these proteins (FDR = 1.25×10^−9^), indicating that common ZIKV/CHIKV interacting proteins may be necessary across many viruses (Fig. 6). Similarly, we identified several immune response proteins (FDR = 5.89×10^−8^) (Fig. 6). A significant number of nuclear proteins, including many splicing factors (FDR = 4.00×10^−8^) and proteins found in the nuclear periphery (FDR = 3.73×10^−11^), were identified for both viruses despite their life cycles taking place outside the nucleus (Fig. 6). We also observed enrichment in methylation (FDR = 1.97×10^−19^), ribonucleoprotein complex formation (FDR = 3.09×10^−12^), and ATP binding (FDR = 6.18×10^−7^) (Fig. 6).

### Stable knockdown of identified proteins impacts viral replication

To validate the hits identified based on the increased detection in the Mango-II pulldown experiments, we selected proteins of interest (POIs) involved in RNA binding or known to be involved in viral functions. We selected proteins unique to ZIKV (CTNND1, PABPC1, PACSIN2, PUM2, QARS, TJP2, YTHDF1, RBM47, ARID3A) or CHIKV (DDX5, DDX21, TMPO, COPA, TUBB3, MATR3, FXR1) as well as proteins found in both datasets (BRD4, EIF4G1, FASN, PLEC, SFPQ).

**Figure 7:**
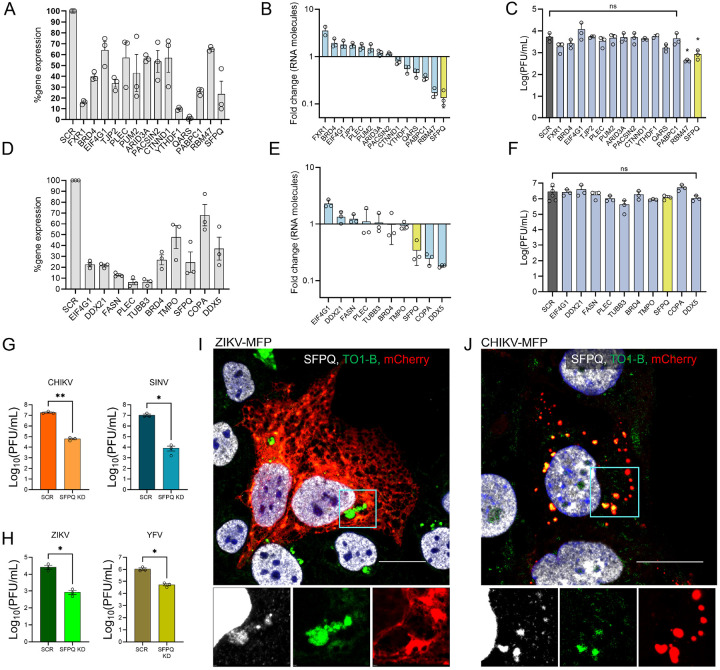
Protein of interest knockdown and screening reveal a significant impact of host factor SFPQ. (A) qRT-PCR measuring RNA corresponding to proteins of interest in JEG-3 KD cell lines reported as a percentage relative to RNA detected in SCR-JEG-3 cells (n=3; error bars represent mean ± SEM). (B) ZIKV vgRNA detected by qRT-PCR from supernatants of infected JEG-3 KD cell lines reported as fold change relative to ZIKV vgRNA detected in supernatant of ZIKV infected JEG-3 SCR cells (n=3; error bars represent mean ± SEM). (C) Viral titers determined by plaque assay of supernatant from ZIKV infected JEG-3 KD cells (n=3; error bars represent mean ± SEM, significance determined by One-way ANOVA, n.s. p > 0.05, *p< 0.01). (D) qRT-PCR measuring RNA for proteins of interest in U2OS KD cell lines reported as a percentage relative to RNA detected in U2OS SCR cells (n=3; error bars represent mean ± SEM). (E) CHIKV vgRNA detected by qRT-PCR from supernatants of infected U2OS KD cell lines reported as fold change relative to CHIKV vgRNA detected in supernatant of CHIKV infected U2OS SCR cells (n=3; error bars represent mean ± SEM). (F) Viral titers determined by plaque assay of supernatant from CHIKV infected U2OS KD cells (n=3; error bars represent mean ± SEM, significance determined by One-way ANOVA, n.s. p > 0.05). (G) Viral titers collected at 24 h.p.i. from scramble (SCR) or SFPQ knockdown JEG-3 cells infected with CHIKV (left) and SINV (right). (n=3; error bars represent mean ± SEM, significance determined by Student’s T test). (H) Viral titers collected at 24 h.p.i. from scramble (SCR) or SFPQ knockdown JEG-3 cells infected with ZIKV (left) and YFV (right). (n=3; error bars represent mean ± SEM, significance determined by Student’s T test). (I-J) Fluorescent micrographs of ZIKV-MFP (I) or CHIKV-MFP (J) infected JEG-3 cells treated with TO1-B (green) and SFPQ antibody (far-red, pseudo-colored white). The red channel shows mCherry-tagged viral proteins ZIKV NS2A (I) or CHIKV nsP3 (J). The cyan box indicates ROI. An expanded image of ROI in separated far-red, green, and red channels is shown below. All scale bars correspond to 20 μm. See also Fig. S3.

To determine the impact of vgRNA-interacting proteins on viral infection, we depleted POIs from human cells through shRNA-driven knockdown. For ZIKV, stable cell lines were generated in JEG-3 cells. Relative gene expressions were evaluated by qRT-PCR, which confirmed all knocked-down (KD) cell lines showed a reduction of ≥ 35% as compared to the scrambled (SCR) control (Fig. 7A). These stable cell lines were infected with ZIKV, and cell lysates were collected at 36 h.p.i. to determine ZIKV vgRNA levels by qRT-PCR (Fig. 7B). Knockdown of FXR1 increased ZIKV RNA significantly with a fold change (FC) of 2.01 (Fig. 7B). Knockdown of EIF4G1, BRD4, TJP2, PLEC, and PUM2 each increased the RNA copy number relative to SCR more than 1.45-fold as determined by qRT-PCR demonstrating a significant antiviral effect (Fig. 7B). In contrast, YTHDF1, QARS, and PACSIN2 knockdown cell lines resulted in a decrease in RNA copy number relative to SCR, with fold change values greater than 0.72 (Fig. 7B). Knockdown of ARID3A, BRD4, CTNND1, and FASN had a minimal impact on vgRNA production, with RNA fold change values of1.19, 1.17, 0.92, and 0.83, respectively (Fig. 7B). Finally, the knockdown of PABPC1, RBM47, and SFPQ was the most proviral with FC < 0.5 (Fig. 6B). The most substantial effect was caused by the depletion of SFPQ in JEG-3 cells (FC = 0.13) (Fig. 7B). In addition to evaluating RNA copy numbers by qRT-PCR, we also tested infectious virus production. Cell culture supernatants were collected at 20 h.p.i. and virus titers were determined by plaque formation assay (Fig. 7C). ZIKV in SCR-JEG-3 cells produced a titer of5.33 ×10^3^ PFU/ml at 20 h.p.i. We observed a slight increase in titer from EIF4G1-KD cells (1.20×10^4^ PFU/ml). Out of the 14 total KD cell lines tested, the most significant impact was observed with the depletion of RBM47 (4.26×10^2^ PFU/ml) and SFPQ (8.53×10^2^ PFU/ml).

To evaluate the importance of the POI identified by CHIKV vgRNA pulldown, knockdown cell lines were generated in U2OS cells and infected with CHIKV. Due to the faster life cycle of alphaviruses, supernatant for infectious CHIKV production determination was collected at 8 h.p.i., and total RNA was purified from cell lysate at 16 h.p.i. for qRT-PCR. All knockdown cell lines showed a reduction in RNA of over 30% for the POI (Fig. 7D). Depletion of EIF4G1 reduced the RNA copy number by more than 2-fold as compared to SCR- U2OS cells, indicating a strong antiviral effect Fig. 7E). We also observed an antiviral effect for DDX21 (FC = 1.3) and FASN (FC = 1.2) knockdown cells (Fig. 7E). Knockdown of FXR1, TUBB3, PLEC, TMPO, and BRD4 caused minimal impact with fold change values ranging from 0.87–1.02 (Fig. 7E). SFPQ-KD was proviral, much like ZIKV, with a reduction in RNA copy number (FC = 0.32) (Fig. 7E). The most substantial proviral impact was seen in the depletion of COPA and DDX5 with FC values of 0.24 and 0.18, respectively. Finally, titer determination by plaque assay was performed on the supernatant from infected knockdown cells collected at 8 h.p.i. The titer of WT CHIKV in U2OS-SCR cells was (2.86×10^6^ PFU/ml). SFPQ-KD in U2OS cells resulted in a slight reduction (1.20×10^6^ PFU/ml). We also observed a decrease in viral titer in TUBB3 (4.33×10^5^ PFU/ml) and TMPO (8.80×10^5^ PFU/ml) knockdown cells.

### SFPQ colocalizes with vgRNA in ZIKV- and CHIKV-infected cells

Due to the significant impact on RNA levels in both viruses and the reduction in titer for ZIKV in JEG-3 cells, we selected SFPQ for further characterization. To determine if SFPQ knockdown would impact alphavirus titers when infected in JEG-3 cells, we infected JEG-3-SCR or JEG-3-SFPQ-KD cells with CHIKV and SINV and harvested the virus-containing supernatant at 24 h.p.i. (Fig. 7G). Both viruses showed a significant decrease in viral titer of ~ log and ~ 3 log PFU/ml in the knockdown cells compared to SCR cells, indicating that SFPQ is necessary for alphavirus production during infection in JEG-3 cells. To test whether the knockdown of SFPQ in JEG-3 cells impacted other orthoflaviviruses, we infected JEG-3-SCR or JEG-3-SFPQ-KD cells with YFV as well as ZIKV as a control and harvested virus-containing supernatant at 24 h.p.i. (Fig. 7H). In both cases, we saw a significant decrease of ~ 1.5 log PFU/ml in virus produced in the SFPQ knockdown cells compared to SCR cells, although the difference in titer was greater for ZIKV. Growth kinetic analysis was performed in SCR and SFPQ-KD JEG-3 cells for ZIKV, YFV, CHIKV, and SINV (Figure S2 E-H). ZIKV and YFV showed a greater reduction in titer at earlier time points (Figure S2 E-F). In comparison, CHIKV and SINV showed greater reduction in titer at late time points (Figure S2 G-H), indicating different functionality of the vgRNA-SFPQ interaction in flaviviruses vs. alphaviruses.

Next, we evaluated the intracellular localization of SPFQ with viral RNA and proteins using an immunofluorescence assay (IFA). Glass-bottomed dishes were seeded with JEG-3 cells and infected at a multiplicity of infection (MOI) of 0.1 with either ZIKV-MFP or CHIKV-MFP. The cells were then fixed at 36 h.p.i. or 16 h.p.i. respectively and treated with an antibody targeting SFPQ. Being a predominantly nuclear protein, in both ZIKV-MFP and CHIKV-MFP-infected cells (Fig. 7I–J) and uninfected cells (Figure S3 A), a majority of SFPQ was detected in the nucleus. In ZIKV-MFP, a small amount of SFPQ was detected in the perinuclear region, colocalizing with TO1-B stain, indicating an interaction with vgRNA (Fig. 5G). A similar relocalization of SFPQ was observed in ZIKV-MFP-infected JEG-3-SFPQ-KD cells, indicating that even with decreased SFPQ levels, the available SFPQ is still capable of interacting with the virus (Figure S2). In cells infected with CHIKV-MFP, SFPQ was observed to colocalize with vgRNA in nsP3-containing vesicles (Fig. 7J).

### SIM reveals SFPQ-ZIKV vgRNA interaction in high-resolution

**Figure 8:**
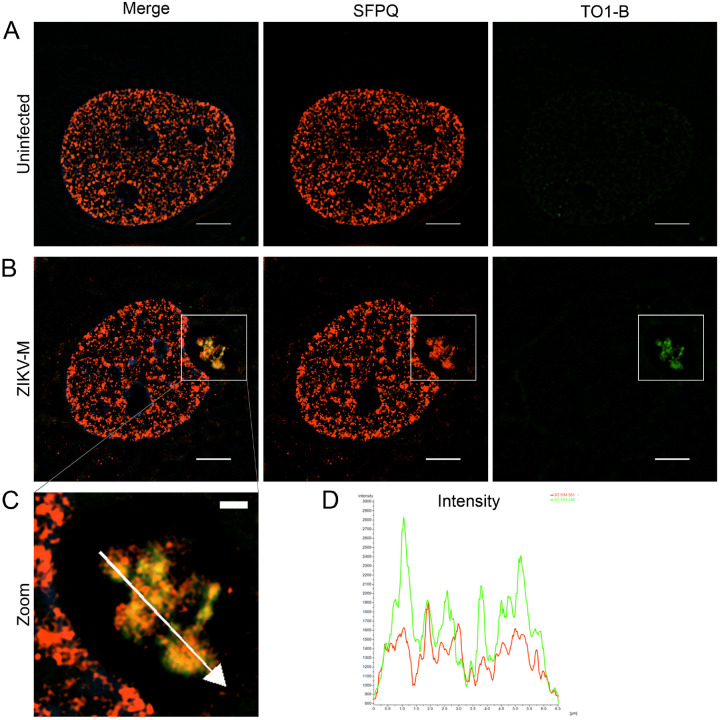
Super-resolution imaging of vgRNA-SFPQ colocalization using SIM. (A-B) Fluorescent micrographs taken by SIM of JEG-3 cells uninfected (A) or infected with ZIKV-M (B), fixed at 36 h.p.i., and treated with anti-SFPQ antibody (red) and TO1-B stain (green). The scale bar in (A-B) indicates 5 μm. (C) Expanded ROI corresponding to white boxes in (B). The scale bar in (C) indicates 1 μm. (D) Intensity graph of green (535 nm) and red (610 nm) fluorescence channels corresponding to the white arrow in (C).

The images obtained by confocal microscopy showed the region of SFPQ-ZIKV vgRNA interaction; however, the resolution (~ 0.2–0.3 μm) is insufficient to show details within this perinuclear structure. We extended our imaging analysis by performing structured illumination microscopy (resolution ~ 120 nm) to further examine this phenomenon. In uninfected cells, SFPQ is observed exclusively in the nucleus (Fig. 8A). In ZIKV-M-infected cells, we observed perinuclear structures morphologically similar to those seen by confocal microscopy containing SFPQ (red) and vgRNA (green) (Fig. 8B). This colocalization is oriented in the center of a kidney-shaped indentation of the nucleus (Fig. 8B), and nuclear reshaping is not observed in uninfected cells (Fig. 8A). Within the observed structure, SFPQ and vgRNA are highly colocalized, with intensity peaks overlapping within < 0.5 um (Fig. 8C–D). This association confirms that ZIKV recruits the proviral host factor SFPQ to the perinuclear region, where it colocalizes with the vgRNA during infection.

The dataset identified here provides information on many vgRNA-binding proteins that interact with CHIKV and ZIKV. Further exploration of proteins involved in both viral families will elucidate the common and unique strategies that distinct positive-strand RNA viruses employ to effectively hijack cellular pathways. The Mango-II aptamer tagging system, applied here to viral RNA, provides a sensitive and robust method for evaluating intracellular spatial and temporal dynamics of viral genomes. Our approach, which incorporates fluorescently tagged viral proteins, provides context for the extensive modifications these viruses induce in infected cells. Most importantly, the dual-action functionality of the Mango-II aptamer tag facilitates the identification of key RNA-binding proteins such as SFPQ, paired with validation by imaging using a single elegantly designed construct, which will provide insights into mechanisms of viral RNA interactions as well as mechanisms of proviral and antiviral strategies.

## Discussion

In this study, we have developed a dual-action approach to investigate viral RNA from both imaging and proteomics perspectives by generating Mango-II aptamer-tagged genomes in both ZIKV and CHIKV. Our dual-labeled viruses were designed to maintain viral function, thus preserving the biologically relevant context of active infection. We showed that the TOI-B stain detects vgRNA but does not detect the dsRNA replication intermediate which is likely due to the unfolding of the Mango-II secondary structure by the viral helicases, NS3 in ZIKV and nsP2 in CHIKV, combined with the base pairing to the negative strand RNA during replication, which would prevent TO1-B binding. Importantly, the differentiation of (+) strand RNA recognition provides a potential method of spatiotemporally distinguishing between replicating RNA from single-stranded translating or packaging viral RNA in infected cells. The stable integrations into live infectious viruses enabled prolonged imaging of a highly detectable fluorogenic aptamer using a superior imaging platform that revealed insights into the dynamics of viral RNA during infection with two distinct (+) single-stranded RNA viruses.

Viral RNA imaging in live cells has been a long-standing challenge in the field due to the limited availability of RNA tools for visualization. Our approach combines a small, state-of-the-art RNA aptamer with established fluorescent protein tagging sites in both ZIKV and CHIKV to visualize the simultaneous intracellular dynamics of viral RNA and proteins. The ZIKV vgRNA was observed mainly on and around the ER, which agrees with its highly membrane-associated life cycle. In contrast, CHIKV vgRNA exhibited more cytoplasmic and vesicular localization, which reflects the known characteristics of translation and replication in alphaviruses. Notably, the comparison of two disparate viruses provides rigorous validation of our results by demonstrating differences in TO1-B localization that are dependent on specific virus infections. Future studies can adapt this system to additional single-stranded RNA virus families to further compare the functions of different viral genomes.

The compatibility of Mango-II with streptavidin pulldown is a significant advantage that we leveraged to identify host proteins that interact with viral RNA in the context of infected cells. We performed RNA pulldown using the Mango-II aptamer tag for ZIKV and CHIKV. The most significantly enriched protein pulled down with ZIKV vgRNA was NS5, which acts as the viral RNA-dependent RNA polymerase (RdRp) and possesses methyltransferase activity responsible for genome capping. We also identified the structural protein prM, which has not been previously shown to interact with vgRNA. Previous work from our lab and others have indicated an essential role for prM in the assembly of immature viral particles, which is supported by the data shown here.^74–77^ In the CHIKV RNA pulldown, non-structural proteins nsP1, nsP2, and nsP3 were detected, all of which are involved in genome replication. Unlike flavivirus NS5, the alphavirus RdRp nsP4 is stoichiometrically less abundant and rapidly degraded which agrees with the absence of nsP4 in our proteomic analysis.^38,78^ The most strongly enriched protein over WT CHIKV was nsP3, which was observed colocalizing with the Mango-II aptamer-tagged vgRNA in large dynamic vesicles in live-infected cells. Additionally, structural proteins E1, E2, and CP were detected, indicating that the vgRNA interacts with these proteins during nucleocapsid core assembly and virus budding. Notably the capsid protein of ZIKV was not identified by our proteomic analysis, likely due to fundamental differences in nucleocapsid core assembly and genome packaging which are not well understood for orthoflaviviruses. The ZIKV capsid-RNA binding mechanism may disrupt the RNA secondary structures in a way that is markedly different from alphavirus nucleocapsid formation.

In addition to viral proteins, we identified 814 host protein interactions for ZIKV and 275 host protein interactions for CHIKV. A cross-reference of this data with previous viral RNA pulldown studies showed appreciable agreement with the literature. One example of an RNA-binding protein identified by ZIKV vgRNA pulldown is RBM47, which was previously demonstrated by RNA-seq to be upregulated in human cells infected with DENV.^79^ In this study, RBM47 knockdown in JEG-3 human placental cells led to a significant decrease in ZIKV production, suggesting a proviral role. Similarly, one RNA binding protein identified by CHIKV pulldown was DDX5 (RNA Dead-box helicase 5). DDX5 was shown to be a proviral factor, relocalizing from the nucleus to the cytoplasm in association with viral RNA in SINV.^80^ More broadly, DDX5 serves a known proviral function by suppressing the IFN response, thereby promoting virus infection in several viruses, including SARS-CoV, HCV, JEV, and IFV.^81^ Here, DDX5 knockdown in U2OS cells reduced virus replication, indicating a proviral effect. Despite having distinctly different life cycles, the vgRNA of ZIKV and CHIKV showed interaction with many of the same proteins, suggesting either similar protein interactions are being hijacked for various purposes or unrelated viruses are using identical strategies in infected cells.

Of the selected host protein interactions identified by Mango-II aptamer pulldown and characterized in this study, SFPQ showed the most significant impact on titer for both viruses. SFPQ is a predominantly nuclear splicing factor and RNA-binding protein that has been shown to interact with positive single-stranded RNA viruses, such as SARS-CoV-2.^82^ Considering ZIKV and CHIKV are neurotropic viruses, it is essential to note that SFPQ is related to neuronal development and has been linked to Alzheimer’s disease as well as amyotrophic lateral sclerosis (ALS) and frontotemporal dementia (FTD).^83,84^ The knockdown of SPFQ in JEG-3 placental cells led to a significant reduction in titer for ZIKV and CHIKV, as well as additional representative members of the *Flaviviridae* and *Togaviridae* families. We also observed the redirection of SFPQ from the nucleus in association with vgRNA to the cytoplasmic, nsP3-containing vesicles in CHIKV-infected cells and the perinuclear region in ZIKV-infected cells, which was further validated by super-resolution imaging. Collectively, our data suggest that SFPQ plays a significant role in the biogenesis of both alphaviruses and orthoflaviviruses through a coordinated interaction with the vgRNA.

This study employs a state-of-the-art aptamer tagging system to investigate the viral genomes of ZIKV and CHIKV. This strategy facilitates complementary assays through both live-imaging and AP-MS that can be used to identify and validate host protein interactions. Future work will combine this approach with other tools, such as the Sun Tag system, to study viral RNA during translation.^85^ The dual-action approach established here has the potential to revolutionize the study of genome interactions and dynamics in diverse RNA viruses.

### Limitations of the study

As evidenced by the relatively low abundance of host proteins detected in the CHIKV-infected U2OS pulldown compared to the ZIKV-infected JEG-3 pulldown, we report here that the Mango-II pulldown protocol does not work equally well in all cell types. One explanation is varying concentrations of biotin in cell growth media which inhibits streptavidin resin binding. The U2OS cells used for CHIKV pulldown and host protein interaction screening also have an attenuated immune response, which may obscure the impact of host protein knockdowns as seen with SFPQ. The results presented here do not inform the mechanism of SFPQ proviral activity, which will require future study.

The imaging results here are insufficient to determine which population of vgRNA is detected. Our current labeling system is specific to the viral RNA and a single viral nonstructural protein for each virus. Future studies are required to track viral structural proteins to study entry, uncoating, and early RNA trafficking during the eclipse period. Live imaging of viral structural proteins has not yet been established for orthoflaviviruses. AP-MS captured a single time-point for each virus eliminating potential interactions exclusive to early or late infection. All SFPQ imaging was performed through immunofluorescence assay which is limited to fixed cells. Addressing these limitations could be achieved by coupling our system with other imaging modalities, including cryoelectron tomography (cryo-ET), correlative light, and correlative light and electron microscopy (CLEM).

## Materials and Methods

### Cell lines and viruses

JEG-3 (ATCC, #HTB-36) cells were maintained in DMEM: Nutrient Mixture F-12 (DMEM/F-12) (Thermo Fisher Scientific, #12400–024). HEK-293T (ATCC, #CRL-1573), VeroE6 (ATCC, #CRL-1586), and Huh 7.5 (a kind gift from Dr. Charles M. Rice, Rockefeller University) were maintained in Dulbecco’s Modified Eagle Medium (DMEM) (Thermo Fisher Scientific, #12800–028). U2OS (ATCC, #HTB-96) cells were maintained in McCoy’s 5A Medium Modified (Sigma Aldrich, #M4892). BHK-15 (a kind gift from Dr. Richard J. Kuhn, Purdue University) was maintained in Minimum Essential Medium (MEM) (Thermo Fisher Scientific #41500–018). All media were supplemented with 10% fetal bovine serum (FBS) (Avantor Seradigm, #97068–085) and Penicillin-Streptomycin antibiotic (Corning Inc., #30–002-CI). All cells were grown at 37°C incubation with 5% CO_2_ unless otherwise noted.

ZIKV MR766 cDNA clones under a CMV promoter were used to produce DNA-launched wild-type and tagged ZIKV.^64^ CHIKV 181/25, SINV (Toto64), and YFV-17D cDNA clones under the SP6 promoter were *in vitro* transcribed and used to produce wild-type and tagged CHIKV, SINV, and YFV, respectively.^65,66^ Transfections were performed in HEK-293Tcells to produce virus stocks of all CHIKV and ZIKV viruses using Polyethylenimine Hydrochloride as the transfection reagent (Polysciences, #49553-93-7). Cell culture supernatants containing ZIKV were collected at 4 days post-transfection, and supernatants containing CHIKV were collected at 24 hours post-transfection.

### Plasmids and cloning

The pLenti-mCherry-Mango-II × 24 plasmid was obtained from Addgene (#127587), and Mango-II triplet inserts were amplified by PCR (Table S1) using Q5 High-Fidelity 2X Master mix (New England Biolabs, M0492S) with primers containing BssHII or BamHI restriction enzyme digestion sites (Table S1) and purified using a 10% acrylamide gel. A BssHII site was introduced into ZIKV cDNA between NS1-NS2A and a BamHI site was introduced into 181/25 cDNA between nsP1_385–386_ (Table S1) by site-directed mutagenesis using Phusion DNA polymerase (New England Biolabs, #MO530), and Mango-II triplet was cloned into the cDNA clone by restriction digestion followed by ligation using T4 DNA ligase (New England Biolabs, 0202S). The cDNA plasmid sequences containing the Mango-II triplet and/or mCherry inserts were confirmed by Nanopore whole plasmid sequencing at the Penn State Genomics Core facility. Resulting sequences corresponding to the final tagged clones are shown in Table S1.

### Plaque assays

Viral titers were determined by plaque assays in Vero E6 cells for all ZIKV constructs and BHK-15 for CHIKV, YFV, and SINV. Cells were plated into 24-well plates one day prior to infection to form monolayers. On the day of infection, media was removed, and virus stocks were diluted in DMEM (ZIKV) or MEM (CHIKV, YFV, and SINV) containing 10 mM HEPES (N-2-hydroxyethylpiperazine-N’−2-ethanesulfonic acid; Sigma Aldrich, #HO887) and pipetted directly onto cells. The infection continued for 1 hr at 37 °C with rocking and was overlaid with 500 μL of growth media containing 1.5% colloidal cellulose (Sigma-Aldrich, #435244) and 1% FBS. Plates were incubated for 6 days (ZIKV) or 4 days (CHIKV, YFV, and SINV). Plaques formed were counted after fixing the cells with neutral-buffered formalin and staining with Crystal violet (Thermo Fisher Scientific, #21932).^74^

### Growth Kinetics

For the Growth kinetics of ZIKV, ZIKV-M, and ZIKV-MFP, Vero E6 cells grown in 6-well plates were infected with the virus at MOI=1.0, and 200 μL of supernatant was collected and replenished with fresh media every 24 hours for 4 days. For wild-type, CHIKV and CHIKV-MFP growth kinetic analysis, BHK-15 cells were infected with the virus at MOI=1.0, and 200 uL of supernatant was collected and replenished with fresh media at 12, 24, 48, and 72 h.p.i. Virus titers were determined by plaque assays as described elsewhere. For growth kinetic analysis in JEG-3 cells of ZIKV, CHIKV, YFV-17D, and SINV (Toto64), cells were infected with the viruses at MOI=0.1. Supernatants (200 μL) were harvested at the time points indicated in the graphs, and virus titers were determined by plaque assays as described above.

### Confocal and super-resolution microscopy

All confocal micrographs and videos were acquired using a Nikon A1R confocal microscope with a heated 60× oil immersion objective and 1.4 numerical aperture. Mention resonance scanners if you have used them. For live imaging, an environmentally controlled microscope stage-top imaging chamber (Tokai Hit, Fujinomiya, Shizuoka Prefecture, Japan) was used to maintain temperature (37 °C) and CO_2_ concentration (5%) conditions. Cells were imaged in either FluoroBrite DMEM (Thermo Fisher, #A1896701) for live-cell imaging or FluoroSave (Millipore Sigma, #345789) for fixed-cell imaging, supplemented with 140 mM KCl (Millipore Sigma, PX1405–1) and 1 mM MgCl_2_ (New England Biolabs, #B0510A) to facilitate aptamer folding. For structured illumination microscopy (SIM) of fixed cells, the motorized inverted Nikon ECLIPSE Ti-E TIRF microscope with the perfect focus system was used with CFI SR Apochromat TIRF 100×oil objective (NA1.49). Laser and emission band-passes used were as follows: blue, excitation of 405 nm and emission of 425–475 nm; green, excitation of 488 nm and emission of 500–550 nm; red, excitation of 561 nm and emission of 570–620 nm; and far-red, excitation of 640 nm and emission of 660–740 nm. Nikon NIS Elements software was used for all image acquisition and analysis. Nonlinear lookup tables (LUTs) were used to adjust brightness and contrast for clarity. Gaussian convolution processing was used to reduce image noise. Videos were processed using ImageJ software^86^

### Mango-II Aptamer Tag Staining and imaging

Cells used for Mango-II imaging were stained in 250 nM TO1-B Mango-II aptamer stain (Applied Biological Materials, #G955). For fixed cells, the stain was diluted in PBS, and for live cells, the stain was diluted in growth media. The diluted stain was supplemented with 140 mM KCl and 1 mM MgCl_2_ to facilitate aptamer folding. Cells were incubated with stain for 45–60 min at 37 °C and washed twice with PBS supplemented with 140 mM KCl and 1 mM MgCl_2_ prior to imaging.

### Fluorescence in Situ hybridization (FISH)

Custom Stellaris^™^ RNA FISH Probes were designed against the viral genomes of ZIKV and CHIKV by using the Stellaris RNA FISH Probe Designer (LGC, Biosearch Technologies, Petaluma, CA) available online at www.biosearchtech.com/stellarisdesigner (version 4.2). Probes set containing 48 unique probes targeting the viral RNA were labeled with Quasar 670 (Biosearch Technologies), following the manufacturer’s instructions available online at www.biosearchtech.com/stellarisprotocols. For imaging, ZIKV-infected JEG-3 cells or CHIKV-infected U2OS cells grown in glass-bottomed dishes (Ibidi, #81218–200) were fixed in 3.7% formaldehyde (VWR, #10790–710) and permeabilized using 70% ethanol according to the Stellaris FISH protocol. Cells were washed with Stellaris Wash Buffer A (LGC Biosearch Technologies, #A-SFM-WAI-60) and then incubated in a hybridization solution containing FISH probes diluted 1:100 in Stellaris Hybridization Buffer (LGC Biosearch Technologies, #SMF-HBI-10). After overnight incubation at 4°C, cells were washed in Stellaris Wash Buffer A for 30 minutes, then stained with Hoechst and TO1-B Mango-II aptamer stain as described elsewhere. Finally, cells were washed with Stellaris wash buffer B (LGC Biosearch Technologies, #SMF-WBI-20).

### Immunofluorescence Assay (IFA)

JEG-3 or U2OS cells were grown to a confluency of 25–50% in glass-bottomed dishes (Ibidi, #81218–200), then infected with ZIKV or CHIKV, respectively, at an MOI of 0.1 and incubated at 37°C and 5% CO_2_ for 48–72 h. At indicated time points, the cells were fixed with 3.7% paraformaldehyde for 15 minutes and permeabilized with 0.5% Triton X-100 diluted in PBS. After permeabilization, cells were washed thrice with PBS and blocked in 10 mg/mL bovine serum albumin (BSA) (Sigma, #A7906) diluted in PBS for 1 hr before adding the primary antibody. The Primary antibodies used were SCICONS anti-dsRNA antibody (J2) (Exalpha #10010500) and Anti-SFPQ antibody [EPR11847] (Cell Signalling, #ab177149). After overnight incubation at 4°C, the primary antibody was removed, and cells were washed thrice with PBS. Cells were then treated with a secondary antibody. IRDye^®^ 680RD Goat anti-Mouse IgG Secondary Antibody (LI-COR, # 926–68070) was used to detect dsRNA, and IRDye^®^ 680RD Goat anti-Rabbit IgG Secondary Antibody (LI-COR, #926–68071) or TRITC Goat anti-Rabbit IgG (H+L) Secondary Antibody (Thermo Fisher Scientific, 31670) to detect SFPQ. Secondary antibodies were diluted 1:200 in 10 mg/mL BSA for 1 h, followed by staining with Hoechst stain and TOI-B Mango-II aptamer stain (Applied Biological Materials #G955) diluted in PBS containing 140 mM KCl and 1 mM MgCl_2_. Cells were subsequently washed thrice with PBS containing 140 mM KCl and 1 mM MgCl_2_ before imaging using confocal or SIM.

### Transmission Electron Microscopy

Huh 7.5 cells were plated on 150 mm culture dishes (Thermo Fisher Scientific, #150468) and grown for 36 h to 100% confluency. Cells were infected with the virus at an MOI of 1.0. After 36 hours for ZIKV and 16 hours for CHIKV, respectively, cells were washed with PBS, followed by 0.1 M sodium cacodylate buffer. Cells were treated with 10 ml of fixation buffer containing 0.1 M sodium cacodylate buffer and 2.5% glutaraldehyde overnight at 4 °C and harvested using a cell scraper. The cell suspension was centrifuged for 10 minutes at 500 ×g at 4 °C. Cell pellets were resuspended in fresh fixation buffer for 60 min on ice, and centrifuged cell pellets were washed twice with 0.1 M Sodium cacodylate buffer, then post-fixed with 1% reduced osmium tetroxide containing 0.8% Potassium ferricyanide and 0.1 M cacodylate for 90 min. After osmium tetroxide treatment, cells were washed twice with 0.1 M Sodium cacodylate buffer and once with water, then stained with En bloc stain containing 2% Uranyl acetate for 45 min. The stain was removed, and samples were dehydrated using a gradient of 50%, 70%, 85%, and finally 95% ethanol on ice. Cells were removed from ice and washed three times with 100% ethanol, followed by three washes with 100% acetonitrile. Samples were then treated with a 50:50 acetonitrile: Epon mixture for 1 h, then 2 times with 100% Epon for 4 hours each. Samples were then placed in fresh Epon and embedded at 60°C overnight. Grids were prepared using a Leica UC6 ultramicrotome and post-stained with Uranyl Acetate and Lead Citrate. Electron microscopy was performed on the prepared grids using a FEI Talos F200C.

### Affinity-purification Mass-Spectrometry

The RNA pulldown protocol was adapted from Panchapakesan 2018.^60^ All buffers were supplemented with 140 mM KCl and 1 mM MgCl_2_. JEG-3 or U2OS cells were grown in 150 mm culture dishes (Thermo Fisher Scientific, #150468) to produce monolayers and infected with wild-type and Mango-II-tagged ZIKV or CHIKV, respectively, at an MOI of 1.0. Infection was allowed to continue at 37 °C for 48 hours for ZIKV and 16 hours for CHIKV. Cells were washed with cold PBS and harvested using a cell scraper. Cells were then resuspended in cold PBS supplemented with Protease Inhibitor Cocktail (Millipore Sigma, #P8340) and sonicated using a microtip (Branson) to lyse the cells. The lysate was clarified by centrifugation at 48,000 RPM for one hour using a TLA 120.2 rotor in an Optima TLX centrifuge (Beckman Coulter). Strep-Tactin Sepharose beads (IBA Lifesciences, #2-1201-002) were prepared according to the manufacturer’s protocol and then derivatized with 5.98 nM TO1–3PEG-Desthiobiotin (Dtb) from Applied Biological Materials, #G7956) for 15 minutes at room temperature. The beads were washed with X once to remove excess Dtb. The supernatant from the clarified sample cell lysate was then incubated with Dtb-derivatized beads for 60 minutes, washed twice with PBS, and resuspended in X ml of wash buffer (IBA Lifesciences, #2-1003-100). The beads were subjected to Mass Spectrometry analysis at the University of Indiana Proteomics Core facility.

### On bead digests of pulldown samples

After washing, the beads were treated with 8 M Urea, 100 mM Tris hydrochloride, pH 8.5, and then reduced with 5 mM tris(2-carboxyethyl)phosphine hydrochloride (TCEP, Sigma-Aldrich Cat No: C4706) for 30 minutes at room temperature to break the disulfide bonds. The resulting free cysteine thiols were alkylated using 10 mM chloroacetamide (CAA, Sigma-Aldrich, Cat No: C0267) for 30 minutes at room temperature (RT), protected from light. Samples were diluted to 2 M Urea with 50 mM Tris, pH 8.5, and proteolytic digestion was carried out overnight at 35 °C with Trypsin/LysC Gold (0.4 μg, Mass Spectrometry grade, Promega Corporation, Cat No: V5072). After digestion, samples were quenched with 0.4% trifluoroacetic acid (TFA, v/v, Fluka Cat No: 91699).

### LC-MS of ZIKV vgRNA pulldown samples

Approximately 1/15th of each IP sample was loaded onto a 5 cm C18 trap column Acclaim^™^ PepMap^™^ 100 (3 μm particle size, 75 μm diameter; Thermo Scientific, Cat No: 164946) followed by a 25 cm EASY-Spray column (Thermo Scientific, Cat No: ES902) and analyzed using a Q-Exactive Plus mass spectrometer (Thermo Fisher Scientific) operated in positive ion mode. Solvent B was increased from 5%−35% over 100 min to 90% over 2 min, back to 3% over 2 minutes (Solvent A: 95% water, 5% acetonitrile, 0.1% formic acid; Solvent B: 100% acetonitrile, 0.1% formic acid). A data-dependent top 15 method was employed, utilizing an MS scan range of 350–2000 m/z, a resolution of 70,000, an AGC target of 3e6, and a maximum IT of 200 ms. MS2 resolution of 17,500, scan range of 200–2000 m/z, normalized collision energy of 30, isolation window of 4 m/z, target AGC of 1e5, and maximum IT of 150 ms. Dynamic exclusion of 10 sec, charge exclusion of 1, 7, 8, >8, and isotopic exclusion parameters were used.

### Sample preparation and Nano-LC-MS/MS of CHIKV vgRNA pulldown samples

Following bead digestion as described elsewhere, samples were acidified with trifluoroacetic acid (TFA, 0.5% v/v) and desalted on Pierce C18 spin columns (Thermo Fisher, Cat No: 89870) using a wash of 0.5% TFA followed by elution in 70% acetonitrile and 0.1% formic acid (FA). Mass spectrometry was performed utilizing an EASY-nLC 1200 HPLC system (SCR: 014993, Thermo Fisher Scientific) coupled to an Exploris 480^™^ mass spectrometer with FAIMSpro interface (Thermo Fisher Scientific). 1/5th of each fraction was loaded onto a 25 cm Aurora column (Ion Opticks) at 350 nL/min. The gradient was held at 5% B for 5 minutes (Mobile phases A: 0.1% formic acid (FA), water; B: 0.1% FA, 80% Acetonitrile (Thermo Fisher Scientific Cat No: LS122500)), then increased from 4–30%B over 98 minutes; 30–80% B over 10 mins; held at 80% for 2 minutes; and dropping from 80–4% B over the final 5 min. The mass spectrometer was operated in positive ion mode, with a default charge state of 2, advanced peak determination, and lock mass of 445.12003. Three FAIMS CVs were utilized (−40 CV, −55 CV, −70CV), each with a cycle time of 1.3 s and with identical MS and MS2 parameters. Precursor scans (m/z 375–1500) were done with an orbitrap resolution of 120000, RF lens% 40, automatic maximum inject time, standard AGC target, minimum MS2 intensity threshold of 5e3, MIPS mode to peptide, including charges of 2 to 7 for fragmentation with 30 sec dynamic exclusion. MS2 scans were performed with a quadrupole isolation window of 4 m/z, 30% HCD CE, 15000 resolution, standard AGC target, automatic maximum IT, and fixed first mass of 110 m/z.

### Data analysis

Data were analyzed using Proteome Discoverer 2.5.0.400 (Thermo Fisher Scientific). A Homo sapiens reference proteome database (UniProtKB/TrEMBL; 78806 sequences downloaded 05132022), plus ZIKV or CHIKV with mcherry sequences plus common laboratory contaminants (73 sequences including streptavidin) was searched using SEQUEST HT. Precursor mass tolerance was set to 10 ppm, and fragment mass tolerance was set at 0.02 Da with a maximum of 3 missed cleavages. A maximum of 3 modifications were allowed per peptide. Dynamic modifications include methionine oxidation phosphorylation on serine, threonine, and tyrosine, and dynamic protein terminus modifications were acetylation, met-loss, and met-loss plus acetylation. Static modifications were carbamidomethylation on cysteines. Percolator false discovery rate (FDR) filtration of 1% was applied to both the peptide-spectrum match and protein levels. Search results were loaded into Scaffold Q+S Software (version 5.2.2, Proteome Software, Inc.) for visualization.

### Generation of lentiviral vectors and stable cell lines

HEK-293T cells were grown to a confluency of 50–75% in DMEM media supplemented with 10% FBS and then cotransfected with pSPAX2 (Addgene, plasmid #12260), pMD2.G (Addgene, plasmid #12259) and the shRNA corresponding to the gene of interest (Millipore Sigma, Mission)(Table S1) in OptiMEM (Thermo Fisher Scientific, #22600–050) at an approximate concentration ratio of 200 ng: 350 ng: 200ng respectively using lipofectamine 2000 (Invitrogen, #11668–019). After overnight incubation, the media were replaced with fresh DMEM supplemented with 10% FBS, and the supernatant containing lentiviruses was harvested at 24 and 48 h.p.t and stored at −80 °C until use.

JEG-3 or U2OS cells were transduced with 50 μl of lentivirus per well and rocked at room temperature for 5 minutes. Plates were then spun at 2000 rpm for 20 min at 20 °C to increase the efficiency of transduction, a technique known as Spinoculation. After overnight incubation, the media were replaced with fresh growth media and incubated for an additional 24 hours. Transduced cells were selected in media containing 1.25 μg/ml puromycin (selection media), which was replaced every 48 h for at least 6 days to obtain stable cell lines

### Quantitative reverse transcription PCR

Stable cell lines were grown to 90% confluency for infection. The knockdown JEG-3 stable cells were infected with ZIKV, and U2OS stable cells were infected with CHIKV. Cell culture supernatants containing released virus particles were collected for plaque assays, and total RNA from cell lysates was harvested from ZIKV-infected JEG-3 cells at 36 h.p.i. and from CHIKV-infected U2OS cells at 16 h.p.i. RNA was purified using a Quick-RNA miniprep kit (Zymo, #ZR1055) from cell lysates to generate total cDNA using the High-Capacity cDNA Reverse Transcription Kit (Thermo Fisher Scientific, #4374967). The qRT-PCR reaction was prepared in triplicate using PowerUp^™^ SYBR^™^ Green Master Mix (Thermo Fisher Scientific, #A25742) for each sample. Primers aligning to the shRNA target gene, the ZIKV NS2A gene, the CHIKV nsP1 gene, and the host beta-actin gene were used to determine the relative intracellular RNA levels (Table S1). Knockdown efficiency and change in viral RNA levels were measured using the comparative Ct method (ΔΔCt).

### Western blot

Stable cell lines were grown in 6-well plates to 100% confluency and lysed using 250 μL of 1X buffer (LI-COR, #928–40004). Cell lysates were run on SDS-PAGE and then transferred to the nitrocellulose membrane (BioRad #162–0115). After the transfer was complete, membranes were allowed to dry overnight, then rehydrated in TBST (10 mM Tris, 100 mM NaCl, 0.1% Tween 20) and blocked using LI-COR blocking buffer (#927–60001) for 1 hr. Primary antibodies targeting SFPQ (Cell Signaling, #ab177149), and control β-actin Mouse mAb #3700 (Cell Signaling, #8H10D10) were diluted in LI-COR buffer supplemented with 0.2% Tween-20 and incubated with membranes overnight with gentle rocking at 4 °C. Membranes were then washed 3 times in TBST and incubated with IRDye^®^ 800CW Goat anti-Rabbit Ig, (LI-COR, #827–08365) and IRDye^®^ 680RD Goat anti-Mouse IgG (LI-COR, #926–68070) secondary antibodies diluted in LI-COR buffer supplemented with 0.2% Tween-20 for 45 minutes with gentle rocking at room temperature. Membranes were washed again in TBST and then imaged using the LI-COR Odyssey F series 10-channel image scanner.

## Supplementary Material

Supplementary Files

This is a list of supplementary files associated with this preprint. Click to download.

- RNApaperSupplementalfigures06.26.docx
- Supplementalfigure1.jpg
- Supplementalfigure2.jpg
- Supplementalfigure3.jpg
- Supplementaltable1.xlsx
- Supplementaltable2.xlsx
- Supplementaltable3.xlsx
- AZIKVMFPVideo.avi
- BZIKVMFPVideo.avi
- CCHIKVMFPVideo.avi
- DCHIKVMFPVideo.avi
